# The Effect of Fetal and Childhood Growth over Depression in Early Adulthood in a Southern Brazilian Birth Cohort

**DOI:** 10.1371/journal.pone.0140621

**Published:** 2015-10-15

**Authors:** Christian Loret de Mola, Luciana de Avila Quevedo, Ricardo Tavares Pinheiro, Helen Gonçalves, Denise Petrucci Gigante, Janaína Vieira dos Santos Motta, Fernando C. Barros, Bernardo Lessa Horta

**Affiliations:** 1 Postgraduate Program in Epidemiology, Federal University of Pelotas, Pelotas, Brazil; 2 Postgraduate Program in Health and Behavior, Universidade Católica de Pelotas, Pelotas, Brazil; McMaster University, CANADA

## Abstract

**Background:**

Poor nutrition and growth during fetal life and childhood might be associated with depression in adulthood; however, studies evaluating these associations present controversial results, especially when comparing studies using different proxies for fetal growth. We evaluated the association of fetal and childhood growth/nutrition with depression, in adulthood, using different approaches and measurement methods.

**Method:**

In 1982, hospital births (n = 5914) in Pelotas, southern Brazil, were examined and have been prospectively followed. At 30 years, the presence of major depression and depressive symptoms severity was evaluated using the Mini International Neuropsychiatric Interview (MINI) and Beck Depression Inventory (BDI-II). The present study assessed their association with birth weight, premature birth, small for gestational age (SGA), stunting and conditional growth during childhood.

**Results:**

At 30 years, 3576 individuals were evaluated and 7.9% had major depression. Low birth weight (PR = 1.01 95%CI [0.64–1.60]), having been born SGA (PR = 0.87 95%CI [0.64–1.19]) and premature birth (PR = 1.22 95%CI [0.72–2.07]) were not associated with major depression in multivariable models. However, those born SGA who were also stunted in childhood had a higher prevalence of major depression (PR = 1.87 95%CI [1.06–3.29]) and greater odds of scoring a higher level of depression in the BDI-II (OR = 2.18 95%CI [1.34–3.53]).

**Conclusion:**

In this Brazilian cohort of young adults, those born SGA who were also stunted during childhood had a higher risk of depression in adulthood. Our results show that the effect of growth impairment on depression is cumulative.

## Introduction

It has been suggested that exposure to poor nutrition during fetal life, and consequently restricted fetal growth, may be associated with the development of non-communicable diseases (NCD), including mental disorders such as depression. [1, 2] Poor nutrition would create a stressful environment during fetal life, which would increase secretion of glucocorticoids and permanently alter the hypothalamus–pituitary–adrenal axis (HPA), with lifelong effects on neuroendocrine function. [3] In addition intrauterine growth restriction could result in impaired neurodevelopment and neurogenesis, [4] and hippocampal atrophy [5] probably due to cortisol negative feedback during pregnancy. [3] All of these biological alterations have been associated with major depression. [6]

Furthermore, Brown *et al* [7] and Roseboom *et al* [2], showed that individuals born to mothers exposed to the Dutch hunger winter in 1944–45, had an increased risk of developing affective disorders and depressive symptoms in adulthood. Suggesting that fetal undernutrition would be associate to later mental illness.

Most studies that evaluated the effect of fetal adversities on mental health, have assessed the effect of birth weight, and few have used measures like head circumference, length, birth ponderal index or intrauterine growth. [8]

Birth weight depends on gestational age and fetal growth, based on the thrifty phenotype hypothesis [9] only the later would be an important predictor of later disease, product of an stressful fetal environment, which would produce an overstimulation of the HPA, increasing exposure to glucocorticoids. However, it has been shown that prematurity could be associated with impaired neurodevelopment, [4] reason why it is important to disentangle the effect of these exposures.

A recently published meta-analysis reported that most studies have evaluated the association between fetal growth restriction and later depression used birth weight as a proxy. However, few have assessed the effect of been born small for gestational age (SGA), a better proxy for intrauterine growth retardation. This meta-analysis showed that depression might be associated with low birth weight (pooled OR was 1.39 [95% CI 1.21–1.60]); because, few studies included in this meta-analysis evaluated the association of depression with premature birth or SGA, the authors were unable to draw any strong conclusion. [1]

In addition, poor nutrition and growth in early childhood might also program the development of mental disorders. [10–15] It has been shown that poor nutrition and growth during childhood is associated with impaired cognitive development, [16] and that this could be an important predictor of mental health outcomes. [17] In addition, stunting could have neurodegenerative consequences with long lasting effects on neurodevelopment, and predispose individuals to develop different mental disorders. [10, 18, 19] However, few studies have evaluated the association of growth or nutrition status in the first years of life with later depression.

This study aimed at evaluating the association of depression at 30 years, with poor growth during fetal life and childhood, specifically during the first four years of life, in a birth cohort from southern Brazil.

## Material and Methods

### Design, Participants and Setting

In 1982, the maternity hospitals in Pelotas, a southern Brazilian city, were visited daily, during the whole year. We identified 99.2% of all births in the city, and the 5914 live births whose families lived in the urban area of the city were weighed using calibrated pediatric scales and their mothers interviewed on sociodemographic and health related variables by a trained interviewer. These individuals have been followed up on several occasions and further details on study methodology have been published elsewhere. [20]

The following variables were measured at birth: gestational age, estimated from the last menstrual date; family income; maternal age; marital status; maternal schooling; pregnancy risk factors; prenatal visits; type of delivery; child’s sex; number of siblings.

In 1984 and 1986, at a mean age of two and four years, we managed to trace 87% and 84% of the cohort members, respectively. In these visits, children were weighed using calibrated pediatric scales and length/height was measured using a portable stadiometer. Mothers or caregivers were interviewed on sociodemographic and health related variables by a trained interviewer. From February 2012 to February 2013, we tried to locate the entire cohort, at a mean age of 30.2 years, using multiple strategies. Initially, the subjects were sought at their last known address and if they were not located, we searched in existing databases (university databases, telephone directories and social media). Cohort members were invited to visit our Research Clinic, where trained personal interviewed and examined them. The visit included a general interview, physical evaluations and a psychological interview by trained psychologists. We located 4534 cohort members, of these, 3701 were interviewed, 467 were living far from Pelotas, 86 refused and another 280—although not having openly refused—did not attend the clinic in spite of repeated invitations. [21]

### Variables assessed

#### Outcomes

We evaluated mental health outcomes at 30 years using the Mini International Neuropsychiatric Interview (MINI) V5.0, [22] which has been validated for Brazil [23] and allows computation of scores for several psychiatric diagnosis, including major depression and manic/hypomanic episode. We considered as cases of major depression, those individuals who were positive for an episode of depression during the past 2 weeks and negative for a lifetime episode of hypomania or mania. In addition, we used the Beck Depression Inventory (BDI-II) to assess the intensity of depressive symptoms. Those individuals whose BDI-II score was between 0 and 13 points were considered as minimal / no depression, mild depression was defined by a score of 14 to 19, moderate from 20 to 28 and severe from 29 to 63 points. [24]

#### Main exposures

We used the Williams reference population to calculate birth weight for gestational age in z-scores. [25] We considered small for gestational age (SGA) those individuals whose birth weight, according to gestational age and sex, was more than 1.28 standard deviations (SD), below the mean in the Williams reference. [25] All other individuals were considered, as been born adequate-for-gestational age (AGA). Using the World Health Organization (WHO) growth reference curves, [26] z-scores according to age and sex were also calculated for weight and height/length at two and four years. We considered as stunted those children whose length/height for age z-score was more than two SD below the mean. [27]

To evaluate growth from birth to four years, we used conditional growth models. Conditional height is the residual from regressing present height on previous height and weight, and birth weight. Conditional relative weight is present weight regressed on present height, previous height and weight, and birth weight. Therefore, conditional weight at two years was derived from a regression of length at two years and birth weight. Conditional variables measure the child’s deviation of the expected size based on the previous measures and the growth in its own population or a reference population. In other words, if for example, a child has a positive value for conditional height it means that he is taller than expected in view of previous size and thus had a faster rate of linear growth. Having a negative conditional growth would represent the opposite. By using this approach, we avoided collinearity between weight or length gain in subsequent age ranges and regression to the mean. [28]

In addition, later depression could be a consequence of cumulative disadvantages during the first years of life. Therefore, we tested for interaction between been born SGA and nutritional status, and created a new variable combining SGA and stunting at two and four years. This variable had four categories; (i) never stunted nor born SGA; (ii) not born SGA but stunted at two and/or four years; (iii) born SGA but never stunted at two and/or four years; and (iv) SGA and stunted at two and/or four years

### Statistical Analysis

Chi-squared test was used to compare proportions and Poisson regression with robust adjustment of the variance in crude and multivariable analysis to calculate prevalence ratios (PR), [29] for major depression (MINI diagnosis). For the BDI-II, we calculated odds ratios (OR) using ordinal regression and the Brant test was used to assess the proportional odds assumption.

In multivariable analysis, variables that have been reported as related to nutrition at birth and childhood, and mental health were considered as possible confounders. Models including birth weight, premature birth and SGA were adjusted for maternal age (in years), schooling (total amount of studied years), family income at birth (in minimum wages), self-reported skin color (proxy for socioeconomic status [30]), number of previous gestations, pregnancy risk factors (previous mischarge/stillbirth, gestational diabetes, preeclampsia and current mischarge risk), type of delivery (vaginal or C-section), maternal smoking during pregnancy and sex.

In addition, models using height for age at two and four years, and conditional growth, were adjusted for all previously described confounders, birth weight and assets index at two years, mother reference of ‘nerve’ problems (this was used as a proxy of maternal mental distress), father live together, father history of psychiatric illness, parent’s alcoholism and total duration of breastfeeding (in months). We included all these variables, because they could directly affect growth in childhood, and the risk of developing a mental disorder later in life.

Height for age at 4 years was not adjusted for height for age at 2 years, because these variables are collinear. This is why we used conditional growth analysis, which, as explained before, takes into account previous periods of growth and collinearity is not an issue.

Each exposure was evaluated separately. Therefore, we had three models with perinatal variables (birth weight, SGA and premature birth), two models with data on height for age at two and four years, four models evaluating conditional growth and one model evaluating the combined effect of SGA and stunting, having in total 20 models of analysis, 10 for each outcome.

We tested for interactions in fully adjusted models, between SGA, and stunting at two and four years, and between SGA and conditional growth. In addition, we tested for interactions between sex and the main exposures. For statistical analysis, we used STATA v12.0.

### Ethics Statement

We obtained ethical approval for the study from the ethics committee of the ‘Universidade Federal de Pelotas’, all participants signed an informed consent.

## Results

In 2012/2013, 3701 individuals were interviewed, which added to the 325 members know to have died (N = 4026), represented a follow-up rate of 68.1%. Without including deaths follow-up rate would be 62.6%. We evaluated mental health outcomes in 3576 individuals.

The follow-up rate at 30 years was higher among females, those whose mothers had between 5 to 8 years of schooling and whose family income ranged from 1–6 minimum wages and individuals with lower birth weight. Among those subjects who were interviewed at 30 years, 96.6% of them participated in the psychological evaluation; individuals who did not participate were similar to those who did, in terms of socio-demographic and biological characteristics at birth and during childhood (S1 Table).

Prevalence of major depression was 7.9%. In addition, prevalence of mild, moderate and severe depression, using the BDI-II score, was 10.9%, 7.8% and 5.2%, respectively (Table 1).

**Table 1:** Distribution of the 1982 Pelotas birth cohort, according to sociodemographic factors, gestational age, nutritional status at birth, 2 and 4 years, and Mental Health outcomes at 30 years.

	N	Mean (SD)	Prevalence %
Female	1859		52.0
Skin color White	2455		75.1
Mother's with four or less years of schooling	1147		32.1
Mother's age at birth	3577	26.0 (6.2)	
Birth weight (g)	3577	3222 (526)	
Gestational age (weeks)	2885	39.4 (1.8)	
SGA	410		14.2
Stunting 2 years	416		12.7
Stunting 4 years	337		10.5
Major Depression (MINI)	282		7.9
BDI-II			
Mild	388		10.9
Moderate	279		7.8
Severe	187		5.2

SD = standard deviation

In the studied population, 52.0% were women and 32.1% of the mothers had four or less years of schooling at delivery. Mean birth weight and gestational age were 3222 g and 39.4 weeks, respectively. Prevalence of low birth weight (<2500 g) was 7.2%, preterm birth 5.6% and SGA 14.2%. Prevalence of stunting at two and four years was 12.7% and 10.5%, respectively (Table 1).

Table 2 shows that prevalence of major depression was higher among females and individuals from families of low socioeconomic status. In addition, the prevalence of premature birth was independent of socioeconomic and demographic variables, whereas birth weight and birth weight according to gestational age were negatively associated with socioeconomic status.

**Table 2:** Major depression, low birth weight, premature birth and SGA according to sociodemographic characteristic at birth.

	Low birth weight		Gestational age		SGA		Major Depression	
	<2500 g	N	≤37 weeks	N	<-1.28 SD	N		N
*Sex*	p<0.001*		p = 0.96*		p = 0.30*		p<0.001*	
Male	5.8%	1719	5.6%	1401	15.0%	1401	4.2%	1717
Female	8.5%	1858	5.6%	1484	13.5%	1483	11.2%	1859
*Skin color*	p<0.001*		p = 0.86*		p = 0.002*		p = 0.08*	
Black	8.1%	814	5.4%	606	15.5%	606	9.2%	813
White	6.4%	2455	5.6%	2023	13.6%	2022	7.3%	2455
*Family income at birth in minimum wages*	p<0.001		p = 0.29		p<0.001		p<0.001	
≤ 1	11.9%	698	6.0%	504	19.0%	504	10.8%	696
> 1–3	6.3%	1760	5.4%	1416	15.3%	1415	7.8%	1761
> 3–6	6.1%	693	5.9%	596	10.9%	596	7.4%	693
> 6–10	6.0%	218	6.2%	194	8.2%	194	5.5%	218
≥ 10	4.7%	191	4.8%	166	9.0%	166	2.6%	191
*Mother Marital status*	p<0.001*		p = 0.74*		p = 0.19*		p = 0.27*	
Married	7.0%	3297	5.7%	2686	14.2%	2685	7.0%	3298
Not married	10.1%	276	5.1%	196	15.3%	196	8.7%	276
*Maternal age at birth*	p<0.001		p = 0.24		p<0.001		p = 0.019	
< 20	10.8%	535	6.4%	392	23.0%	392	9.2%	535
20–29	6.1%	2050	4.8%	1680	13.3%	1679	8.4%	2049
≥ 30	7.6%	991	6.9%	813	11.8%	813	6.2%	991
*Maternal schooling*	p<0.001		p = 0.53		p<0.001		p<0.001	
0–4	7.8%	1147	5.2%	839	15.6%	839	9.4%	1145
5–8	8.0%	1537	6.1%	1262	15.6%	1261	8.0%	1538
9–11	4.3%	395	4.7%	339	10.9%	339	7.3%	395
≥ 12	5.7%	493	5.6%	443	10.2%	443	4.1%	493

*p-values for heterogeneity using chi-squared test. All other p-values for linear trend. All analyzed individuals had information on major depression, however total N may differ for each variable due to missing data. SD = standard deviation. SGA = small for gestational age.

Table 3 shows that prevalence of major depression was higher among low birth weight individuals in relation to those whose birth weight was > 3500 g. However, this association vanished after controlling for confounders. In analyses using the ordinal categories of the BDI-II, low birth weight, stunting at two or four years, and conditional growth from birth to 24 months, were associated with higher odds of scoring a higher level of depression. However, the magnitude of the associations was reduced after controlling for confounders and the confidence intervals included the reference.

**Table 3:** Crude and multivariable models of the association between birth weight, premature birth, small for gestational age, height for age, conditional growth and depression.

	*Major depression*				*BDI-II*			
		Crude		Adjusted		Crude		Adjusted
	N	PR (95%CI)	N	PR (95%CI)	N	OR (95%CI)	N	OR (95%CI)
*Birth weight according to the gestational age (z-score)*		p = 0.71*		p = 0.4*		p = 0.14*		p = 0.87*
< -1.28 SD	410	1.01 (0.76–1.35)	370	0.87 (0.64–1.19)	410	1.09 (0.91–1.29)	371	0.94 (0.78–1.14)
-1.28 / 0 SD	1195	1.09 (0.73–1.62)	1164	0.86 (0.57–1.31)	1285	1.19 (0.93–1.52)	1171	1.07 (0.81–1.40)
> 0 SD	1277	1	1086	1	1206	1	1097	1
*Gestational age in weeks*		p = 0.42**		p = 0.46**		p = 0.99**		p = 0.94**
≤ 37	162	1.24 (0.74–2.08)	147	1.22 (0.72–2.07)	163	1 (0.70–1.42)	148	0.99 (0.67–1.45)
> 37	2721	1	2474	1	2740	1	2492	1
*Birth weight*		p = 0.42*		p = 0.13*		p = 0.006*		p = 0.5*
< 2500 g	258	1.55 (1.02–2.37)	223	1.01 (0.64–1.60)	258	1.69 (1.28–2.24)	223	0.91 (0.75–1.11)
2500 / 3000 g	965	0.91 (0.65–1.27)	888	0.64 (0.45–0.92)	967	1.08 (0.89–1.31)	891	0.78 (0.63–0.97)
3000 / 3500 g	1312	1.11 (0.82–1.49)	1195	0.99 (0.73–1.35)	1315	1.05 (0.88–1.25)	1200	1.21 (0.88–1.67)
> 3500 g	1040	1	952	1	1048	1	960	1
*Height for age in z-score at 2 years*		p = 0.3*		p = 0.94*		p = 0.001*		p = 0.42*
< -2 SD	417	1.32 (0.89–1.94)	333	1.06 (0.64–1.76)	410	1.56 (1.22–1.99)	328	1.24 (0.91–1.70)
-2 / 0 SD	1937	0.98 (0.73–1.31)	1603	0.92 (0.64–1.31)	1949	1.11 (0.93–1.32)	1617	0.9 (0.73–1.11)
> 0 SD	919	1	772	1	928	1	781	1
*Height for age in z-score at 4 years*		p = 0.24*		p = 0.6*		p = 0.001*		p = 0.33*
< -2 SD	337	1.37 (0.91–2.08)	276	0.89 (0.52–1.55)	328	1.85 (1.41–2.41)	271	1.29 (0.92–1.79)
-2 / 0 SD	1986	1.02 (0.76–1.38)	1680	0.87 (0.61–1.24)	1998	1.19 (1.00–1.43)	1692	0.95 (0.77–1.18)
> 0 SD	883	1	752	1	894	1	763	1
*Conditional Growth*								
Birth– 24m (Height)	2637	0.91 (0.79–1.06)	2190	1 (0.84–1.20)	2656	0.84 (0.77–0.92)	2208	0.9 (0.81–1.00)
24m - 48m (Height)	2450	0.99 (0.85–1.15)	2186	1.09 (0.93–1.27)	2469	0.94 (0.87–1.03)	2204	1.05 (0.96–1.15)
Birth - 24m (Weight)	2636	0.99 (0.88–1.12)	2189	0.96 (0.83–1.11)	2655	0.98 (0.90–1.07)	2207	0.95 (0.86–1.05)
24m - 48m (Weight)	2448	0.95 (0.83–1.08)	2184	0.95 (0.82–1.11)	2467	1.01 (0.93–1.11)	2202	1.07 (0.97–1.18)
*SGA + Stunting*		p = 0.035**		p = 0.09**		p = 0.004**		p = 0.009**
None	2231	1	1752	1	2252	1	1774	1
Only stunted	152	0.88 (0.46–1.69)	121	0.62 (0.26–1.49)	153	0.92 (0.63–1.34)	122	0.78 (0.50–1.22)
Only SGA	307	1.31 (0.88–1.94)	253	0.93 (0.56–1.55)	302	1.35 (1.05–1.75)	248	1.08 (0.80–1.46)
SGA and Stunted	85	2.1 (1.22–3.63)	67	1.87 (1.06–3.29)	85	1.86 (1.21–2.84)	67	2.18 (1.34–3.53)

*P values for trend analysis.

**p value for heterogeneity.

Models adjusted for skin color, mother’s age, schooling, previous gestations, pregnancy risk factors, C-section, smoking and income at birth. Models including variables at two and four years were adjusted also for assets index, mother ‘nerve’ problems, father live together and history of psychiatric illness, parent’s alcoholism and breastfeeding. PR: prevalence ratio. OR: odds ratio. SD: standard deviation. SGA: small for gestational age.

On the other hand, we tested for an interaction between SGA and stunting at two and four. The interaction term, between SGA and stunting, in the full adjusted model for major depression had a p = 0.042 and for the BDI-II p = 0.008. Compared to those who were neither born SGA nor were ever stunted, individuals born SGA who were also stunted at two or four years, had an increased risk of major depression (PR = 1.87 95%CI [1.06–3.29]) and a higher odds of scoring a higher level of depression in the BDI-II (OR = 2.18 95%CI [1.34–3.53]). (Table 3)

When testing the interaction between SGA and conditional growth, in multivariable models, we found that among those born SGA (N = 189) conditional height from birth to 24 months tended to reduce the risk of major depression (PR = 0.76 95%CI [0.37–1.55]), but the confidence interval included the reference. Among those born adequate-for-gestational age (AGA), the prevalence ratio was 1.04 (95%CI [0.86–1.26). We found a similar pattern for the BDI-II, among those born SGA, conditional height from birth to 24 months reduced the proportional odds (OR = 0.66 95%CI [0.42–1.05]), but not among those born AGA (OR = 0.93 95%CI [0.83–1.03]). For conditional height from 24 to 48 months, differences between those with and without SGA were minimal, same as for conditional weight from birth to 24 and 48 months. In addition, sex did not modify the effect of any of the exposures (all p-values >0.2).

S2 and S3 Tables show that sex and maternal sociodemographic characteristics at birth were important confounders in the association between low birth weight and depression. On the other hand, for stunting at two and four years, sex and maternal demographic characteristics were not important confounders. However, adjustment for socioeconomic characteristics at birth and biological characteristics during pregnancy, decreased, or changed the direction of the associations, and confidence intervals included the reference. Similarly, the main confounders in the association between the combined variable of SGA and stunting were socioeconomic characteristics at birth and biological factors during pregnancy.

## Discussion

In this population that has been prospectively followed since birth, in a southern Brazilian city, we observed that the risk of depression in early adulthood is higher in individuals born SGA who were stunted at age two or four, suggesting a cumulative effect of growth in fetal life and early childhood.

Our results also suggest that, among those who suffered from fetal growth impairment, height gain from birth to 24 months might decreased the risk of depression. However, the confidence intervals included the reference, and we cannot ruled out that this association was by chance. We only had 189 complete case of SGA individuals, so this analysis is underpowered. Nevertheless, these associations should be further explore in future studies.

Based on the thrifty phenotype hypothesis, [9] poor nutrition during fetal life and childhood would overstimulate the HPA axis, increasing exposure to glucocorticoids, producing lifelong effects on neurodevelopment, neurogenesis and hippocampal atrophy. [3–5, 8] In the same token, it has been reported that affective disorders in adulthood, including depression, are associated with low caloric intake by the mother during gestation and stunting in childhood is associated with poor psychological functioning. [2, 7, 8, 10] Nonetheless, these biological pathways could also be related with other mood disorders, like anxiety disorders. [31, 32] Some even suggest that there is an association between fetal growth and comorbid generalized anxiety disorder and major depression, in adulthood, but not with neither of them independently. [33]

We observed in a recently published meta-analysis that adult depression was associated with low birth weight. However, prospective studies, compared to retrospectives, found weaker or non-existing associations between birth weight and depression. [1] Similarly Wojcik *et al* found, in another meta-analysis of birth weight and depression/psychological distress, a small association, but which could be due to publication bias. [34]

In addition, only four studies evaluated the association between depression and SGA, three adjusted the estimates for sociodemographic confounders. Independently of adjustment, three reported increased odds of depression in SGA individuals and one reported a protective effect; however, all confidence intervals included the reference. [1]

On the other hand, few studies have evaluated the relationship between nutritional status or growth in childhood and depression in adulthood. Galler *et al* and Waber *et al* observed that the odds of depression in adolescence was higher among individuals, who suffered from protein energy malnutrition in the first year of life. [15, 35] In addition, Walker *et al* [10] found that stunting was associated with depression like symptoms at 17–18 years, nevertheless this was a cohort study based on an intervention with two stunted groups, and estimates were not adjusted for sociodemographic variables. Because depression and stunting are higher among individuals from low socioeconomic status, the observed association may be due to residual confounding. Indeed, as showed in our results, stunting was associated with depression in crude analysis, but this association disappeared after adjustment for sociodemographic variables. Therefore, our study reinforces the relevance of taking residual confounding by socioeconomic status in the assessment of the evidence of any association between nutrition in the first years of life and depression later in life.

In our study we have found that the effect of fetal and childhood growth over later depression is cumulative. Alford (2013) [14] found similar results to ours, he suggested that low birth weight infants with normal birth length and rapid growth during the first year of life have lower odds of later mental distress. Suggesting that the effect of childhood growth could be modified by fetal growth.

It has been suggested that the first 1000 days of life, from a woman’s pregnancy through her child’s 2nd birthday, is a window of opportunity, where better nutrition can have a life-changing impact. In addition to reducing morbidity and mortality in children, and decreasing the risk of later non-communicable diseases, a better nutrition during this period can have an impact on cognitive, motor, and even socioemotional development, as well as improve schooling and working capacity. [36–38] Our results suggest that a better nutrition during this period may also have an impact on people’s mental health.

On the other hand, the negative results observed in this study, cannot be explained by a higher mortality in individuals with depression due to suicide. In spite of not having specific data on deaths due to suicide, in this cohort, among the 325 deaths that occurred, mortality due to suicide should be extremely low and did not underestimate the association. In a previous report from this cohort, we showed that 244 individuals, died during the first 4 years of life, and 44 between 5–24 years, and only 19 of them died by external causes, mainly violence or accidents. [39]

Concerning selection bias, in order to underestimate an association between low birth weight and depression, attrition rate would need to be related to birth weight and depression. Among low birth weight subjects, follow-up would be lower among subjects with depression, whereas among those with birth weight > 2500 g, attrition rate would be independent of depression. Because such association is unlikely, we believe that our study is not susceptible to selection bias. In addition, non-participation in the psychological interview, among those followed at 30 years, was not related to any of our exposures.

On the other hand, this study could have low statistical power to evaluate the factors associated with major or severe depression because, the prevalence of these outcomes was small. However, most of the associations of birth weight, or stunting with depression disappeared after controlling for confounding by socioeconomic variables. Therefore, we do not believe that lack of association with major depression was due to lack of statistical power.

Residual confounding could be another limitation; however, we have adjusted our estimates for several different measures of sociodemographic, biological and behavioral characteristics in pregnancy, at birth and childhood. Therefore, residual confounding is unlikely. On the other hand, including possible mediators in our adjusted models, could explain the lack of association, due to over adjustment. However, this is unlikely; we only included variables that were antecedent to the exposures in multivariable models.

To our knowledge, this is one of the first studies to evaluate in a birth cohort the association of stunting and childhood growth with depression in adulthood. In addition, we reported results for major depression (MINI), and depression severity (BDI-II) in the same cohort, obtaining very similar estimates and patterns. However, in this study, we have only evaluated one possible mental health outcome, and future research could focus on evaluating if there is an association between fetal and childhood growth, and other mental problems. As mentioned before, several studies have evaluated the association between depression and birth weight, but few have evaluated the association between SGA (a better proxy of fetal growth) and depression in adulthood [1], and almost none have explored the combined effect of SGA and later growth. Therefore, we consider that we still need new studies exploring different possible associations between growth and mental health, evaluating possible casual pathways, testing the impact of mediators and effect modifiers.

## Conclusion

We observed an association between early nutrition or growth, and depression at 30 years, this association seems to be cumulative, because the risk of depression was higher only among subjects who were SGA and were also stunted at age two or four years.

## Supporting Information

S1 Database Supporting database with all variables used for analysis. (DTA)

S1 Table Proportion of individuals from the original 1982 cohort with mental health data in 2012–13, according to selected characteristics. *Chi-squared p-value for heterogeneity between those followed at 30 years and the original cohort. Included 325 members known to have died. **Chi-squared p-value for heterogeneity between those interviewed at 30 years and those with mental health data.
(DOCX)

S2 Table Crude and nested multivariable models of the association between birth weight, premature birth, small for gestational age, height for age and major depression. Model 1 adjusted for sex. Model 2 = Model 1 + skin color, mother’s age at birth. Model 3 = Model 2 + maternal schooling and family income at birth. Model 4 = Model 3 + previous gestations, pregnancy risk factors, C-section, smoking in pregnancy. Model 5 = Model 4 + assets index, mother ‘nerve’ problems, father live together and history of psychiatric illness, parent’s alcoholism and breastfeeding. PR: prevalence ratio. SD: standard deviation. SGA: small for gestational age. NA: not applicable
(DOCX)

S3 Table Crude and nested multivariable models of the association between birth weight, premature birth, small for gestational age, height for age and depression severity using the Beck depression inventory. Model 1 adjusted for sex. Model 2 = Model 1 + skin color, mother’s age at birth. Model 3 = Model 2 + maternal schooling and family income at birth. Model 4 = Model 3 + previous gestations, pregnancy risk factors, C-section, smoking in pregnancy. Model 5 = Model 4 + assets index, mother ‘nerve’ problems, father live together and history of psychiatric illness, parent’s alcoholism and breastfeeding. OR: odds ratio. SD: standard deviation. SGA: small for gestational age. NA: not applicable
(DOCX)
