# Supplementary material for: The Effect of Fetal and Childhood Growth over Depression in Early Adulthood in a Southern Brazilian Birth Cohort
Source: PLoS One. 2015 Oct 15;10(10):e0140621. doi: 10.1371/journal.pone.0140621 (PMC4607416; doi:10.1371/journal.pone.0140621)
Supplement: S1 Table — *Chi-squared p-value for heterogeneity between those followed at 30 years and the original cohort. Included 325 members known to have died. **Chi-squared p-value for heterogeneity between those interviewed at 30 years and those with mental health data. (DOCX) [file pone.0140621.s002.docx]

**S1 Table. Proportion of individuals from the original 1982 cohort with mental health data in 2012-13, according to selected characteristics.**

|  | **Original cohort (number)** | **Followed at 30 years*** | **Followed at 30 years with mental health data**** |
| --- | --- | --- | --- |
| Sex |  | p<0.001 | p=0.82 |
| Male | 3037 | 66% | 96% |
| Female | 2876 | 71% | 97% |
| *Birth weight according to the gestational age (z-score)* |  | p=0.12 | p=0.99 |
| < -1.28 SD | 695 | 69% | 97% |
| -1.28 / 0 SD | 2124 | 68% | 96% |
| > 0 SD | 1850 | 71% | 97% |
| Gestational age weeks |  | p=0.07 | p=0.91 |
| ≤ 37 | 294 | 69% | 97% |
| > 37 | 4380 | 75% | 98% |
| Birth weight in grams |  | p=0.015 | p=0.99 |
| < 2500 | 534 | 72% | 97% |
| 2500 / 3000 | 1560 | 70% | 97% |
| 3000 / 3500 | 2195 | 66% | 96% |
| > 3500 | 1620 | 70% | 97% |
| *Height for age in z-score at 2 years* | | p=0.09 | p=0.96 |
| < -2 SD | 687 | 68% | 96% |
| -2 / 0 SD | 2854 | 72% | 97% |
| > 0 SD | 1388 | 71% | 96% |
| *Height for age in z-score at 4 years* | | p=0.73 | p=0.99 |
| < -2 SD | 518 | 72% | 95% |
| -2 / 0 SD | 2939 | 72% | 96% |
| > 0 SD | 1284 | 73% | 97% |
| Family Income at birth (Minimum wages) | | p<0.001 | p=0.40 |
| ≤1 | 1288 | 67% | 95% |
| >1-3 | 2789 | 71% | 97% |
| >3-6 | 1091 | 70% | 96% |
| >6-10 | 382 | 61% | 98% |
| ≥10 | 335 | 60% | 98% |
| Maternal schooling |  | p<0.001 | p=0.99 |
| 0-4 | 1960 | 69% | 88% |
| 5-8 | 2454 | 71% | 91% |
| 9-11 | 654 | 66% | 93% |
| ≥12 | 839 | 63% | 97% |
|  | | | |

*Chi-squared p-value for heterogeneity between those followed at 30 years and the original cohort. Included 325 members known to have died. **Chi-squared p-value for heterogeneity between those interviewed at 30 years and those with mental health data.
