# Supplementary material for: The Effect of Fetal and Childhood Growth over Depression in Early Adulthood in a Southern Brazilian Birth Cohort
Source: PLoS One. 2015 Oct 15;10(10):e0140621. doi: 10.1371/journal.pone.0140621 (PMC4607416; doi:10.1371/journal.pone.0140621)
Supplement: S2 Table — Model 1 adjusted for sex. Model 2 = Model 1 + skin color, mother’s age at birth. Model 3 = Model 2 + maternal schooling and family income at birth. Model 4 = Model 3 + previous gestations, pregnancy risk factors, C-section, smoking in pregnancy. Model 5 = Model 4 + assets index, mother ‘nerve’ problems, father live together and history of psychiatric illness, parent’s alcoholism and breastfeeding. PR: prevalence ratio. SD: standard deviation. SGA: small for gestational age. NA: not applicable (DOCX) [file pone.0140621.s003.docx]

**S2 Table. Crude and nested multivariable models of the association between birth weight, premature birth, small for gestational age, height for age and major depression.**

|  | **Crude** | **Model 1** | **Model 2** | **Model 3** | **Model 4** | **Model 5** |
| --- | --- | --- | --- | --- | --- | --- |
|  | **PR (95%CI)** | **PR (95%CI)** | **PR (95%CI)** | **PR (95%CI)** | **PR (95%CI)** | **PR (95%CI)** |
| *Birth weight according to the gestational age (z-score)* |  |  |  |  |  |  |
| < -1.28 SD | 1.01 (0.76 - 1.35) | 1 (0.75 - 1.32) | 0.92 (0.68 - 1.25) | 0.88 (0.65 - 1.19) | 0.87 (0.64 - 1.19) | NA |
| -1.28 / 0 SD | 1.09 (0.73 - 1.62) | 1.11 (0.75 - 1.64) | 1.01 (0.66 - 1.53) | 0.93 (0.61 - 1.42) | 0.86 (0.57 - 1.31) | NA |
| > 0 SD | 1 | 1 | 1 | 1 | 1 |  |
| *Gestational age in weeks* | |  |  |  |  |  |
| ≤ 37 | 1.24 (0.74 - 2.08) | 1.24 (0.74 - 2.08) | 1.32 (0.77 - 2.26) | 1.32 (0.77 - 2.24) | 1.22 (0.72 - 2.07) | NA |
| > 37 | 1 | 1 | 1 | 1 | 1 |  |
| *Birth weight* |  |  |  |  |  |  |
| < 2500 g | 1.55 (1.02 - 2.37) | 1.34 (0.88 - 2.04) | 1.17 (0.73 - 1.85) | 1.1 (0.69 - 1.74) | 1.01 (0.64 - 1.60) | NA |
| 2500 / 3000 g | 0.91 (0.65 - 1.27) | 0.8 (0.57 - 1.12) | 0.69 (0.48 - 0.98) | 0.66 (0.46 - 0.94) | 0.64 (0.45 - 0.92) | NA |
| 3000 / 3500 g | 1.11 (0.82 - 1.49) | 1.04 (0.77 - 1.39) | 1 (0.74 - 1.35) | 1 (0.73 - 1.35) | 0.99 (0.73 - 1.35) | NA |
| > 3500 g | 1 | 1 | 1 | 1 | 1 |  |
| *Height for age in z-score at 2 years* | |  |  |  |  |  |
| < -2 SD | 1.32 (0.89 - 1.94) | 1.45 (0.98 - 2.13) | 1.33 (0.87 - 2.03) | 1.07 (0.69 - 1.66) | 0.98 (0.63 - 1.53) | 1.06 (0.64 - 1.76) |
| -2 / 0 SD | 0.98 (0.73 - 1.31) | 1.01 (0.76 - 1.36) | 0.98 (0.71 - 1.34) | 0.89 (0.65 - 1.22) | 0.87 (0.63 - 1.19) | 0.92 (0.64 - 1.31) |
| > 0 SD | 1 | 1 | 1 | 1 | 1 | 1 |
| *Height for age in z-score at 4 years* | |  |  |  |  |  |
| < -2 SD | 1.37 (0.91 - 2.08) | 1.36 (0.90 - 2.06) | 1.32 (0.84 - 2.08) | 1.09 (0.68 - 1.74) | 0.97 (0.61 - 1.55) | 0.89 (0.52 - 1.55) |
| -2 / 0 SD | 1.02 (0.76 - 1.38) | 1.02 (0.76 - 1.37) | 1.07 (0.78 - 1.47) | 0.96 (0.69 - 1.32) | 0.89 (0.64 - 1.24) | 0.87 (0.61 - 1.24) |
| > 0 SD | 1 | 1 | 1 | 1 | 1 | 1 |
| *SGA + Stunting* |  |  |  |  |  |  |
| None | 1 | 1 | 1 | 1 | 1 | 1 |
| Only stunted | 0.88 (0.46 - 1.69) | 0.89 (0.47 - 1.69) | 0.66 (0.30 - 1.45) | 0.66 (0.30 - 1.45) | 0.63 (0.29 - 1.38) | 0.62 (0.26 - 1.49) |
| Only SGA | 1.31 (0.88 - 1.94) | 1.34 (0.91 - 1.99) | 1.32 (0.88 - 1.99) | 1.18 (0.79 - 1.78) | 1.1 (0.73 - 1.66) | 0.93 (0.56 - 1.55) |
| SGA and Stunted | 2.1 (1.22 - 3.63) | 2.22 (1.29 - 3.80) | 2.39 (1.40 - 4.08) | 2.1 (1.21 - 3.63) | 1.83 (1.08 - 3.09) | 1.87 (1.06 - 3.29) |
| Model 1 adjusted for sex. Model 2 = Model 1 + skin color, mother’s age at birth. Model 3 = Model 2 + maternal schooling and family income at birth. Model 4 = Model 3 + previous gestations, pregnancy risk factors, C-section, smoking in pregnancy. Model 5 = Model 4 + assets index, mother ‘nerve’ problems, father live together and history of psychiatric illness, parent’s alcoholism and breastfeeding. PR: prevalence ratio. SD: standard deviation. SGA: small for gestational age. NA: not applicable | | | | | | |
