# Supplementary material for: The Effect of Fetal and Childhood Growth over Depression in Early Adulthood in a Southern Brazilian Birth Cohort
Source: PLoS One. 2015 Oct 15;10(10):e0140621. doi: 10.1371/journal.pone.0140621 (PMC4607416; doi:10.1371/journal.pone.0140621)
Supplement: S3 Table — Model 1 adjusted for sex. Model 2 = Model 1 + skin color, mother’s age at birth. Model 3 = Model 2 + maternal schooling and family income at birth. Model 4 = Model 3 + previous gestations, pregnancy risk factors, C-section, smoking in pregnancy. Model 5 = Model 4 + assets index, mother ‘nerve’ problems, father live together and history of psychiatric illness, parent’s alcoholism and breastfeeding. OR: odds ratio. SD: standard deviation. SGA: small for gestational age. NA: not applicable (DOCX) [file pone.0140621.s004.docx]

**S3 Table. Crude and nested multivariable models of the association between birth weight, premature birth, small for gestational age, height for age and depression severity using the Beck depression inventory.**

|  | **Crude** | **Model 1** | **Model 2** | **Model 3** | **Model 4** | **Model 5** |
| --- | --- | --- | --- | --- | --- | --- |
|  | **OR (95%CI)** | **OR (95%CI)** | **OR (95%CI)** | **OR (95%CI)** | **OR (95%CI)** | **OR (95%CI)** |
| *Birth weight according to the gestational age (z-score)* |  |  |  |  |  |  |
| < -1.28 SD | 1.09 (0.91 - 1.29) | 1.07 (0.90 - 1.28) | 1 (0.83 - 1.20) | 0.96 (0.80 - 1.16) | 0.94 (0.78 - 1.14) | NA |
| -1.28 / 0 SD | 1.19 (0.93 - 1.52) | 1.23 (0.96 - 1.58) | 1.21 (0.93 - 1.56) | 1.14 (0.87 - 1.48) | 1.07 (0.81 - 1.40) | NA |
| > 0 SD | 1 | 1 | 1 | 1 | 1 |  |
| *Gestational age in weeks* | |  |  |  |  |  |
| ≤ 37 | 1 (0.70 - 1.42) | 1 (0.70 - 1.43) | 0.99 (0.68 - 1.45) | 1 (0.68 - 1.47) | 0.99 (0.67 - 1.45) | NA |
| > 37 | 1 | 1 | 1 | 1 | 1 |  |
| *Birth weight* |  |  |  |  |  |  |
| < 2500 g | 1.69 (1.28 - 2.24) | 1.45 (1.09 - 1.94) | 1.36 (1.00 - 1.84) | 1.27 (0.93 - 1.73) | 0.91 (0.75 - 1.11) | NA |
| 2500 / 3000 g | 1.08 (0.89 - 1.31) | 0.94 (0.77 - 1.14) | 0.85 (0.70 - 1.05) | 0.81 (0.65 - 0.99) | 0.78 (0.63 - 0.97) | NA |
| 3000 / 3500 g | 1.05 (0.88 - 1.25) | 0.97 (0.81 - 1.17) | 0.91 (0.76 - 1.11) | 0.9 (0.75 - 1.10) | 1.21 (0.88 - 1.67) | NA |
| > 3500 g | 1 | 1 | 1 | 1 | 1 |  |
| *Height for age in z-score at 2 years* | |  |  |  |  |  |
| < -2 SD | 1.56 (1.22 - 1.99) | 1.78 (1.39 - 2.28) | 1.59 (1.22 - 2.06) | 1.29 (0.98 - 1.70) | 1.17 (0.89 - 1.55) | 1.24 (0.91 - 1.70) |
| -2 / 0 SD | 1.11 (0.93 - 1.32) | 1.17 (0.98 - 1.39) | 1.07 (0.89 - 1.28) | 0.98 (0.81 - 1.18) | 0.94 (0.78 - 1.14) | 0.9 (0.73 - 1.11) |
| > 0 SD | 1 | 1 | 1 | 1 | 1 | 1 |
| *Height for age in z-score at 4 years* | |  |  |  |  |  |
| < -2 SD | 1.85 (1.41 - 2.41) | 1.88 (1.43 - 2.47) | 1.74 (1.31 - 2.31) | 1.38 (1.03 - 1.87) | 1.25 (0.92 - 1.69) | 1.29 (0.92 - 1.79) |
| -2 / 0 SD | 1.19 (1.00 - 1.43) | 1.2 (1.00 - 1.43) | 1.13 (0.94 - 1.37) | 0.99 (0.81 - 1.21) | 0.94 (0.77 - 1.14) | 0.95 (0.77 - 1.18) |
| > 0 SD | 1 | 1 | 1 | 1 | 1 | 1 |
| *SGA + Stunting* |  |  |  |  |  |  |
| None | 1 | 1 | 1 | 1 | 1 | 1 |
| Only stunted | 0.92 (0.63 - 1.34) | 0.93 (0.63 - 1.36) | 0.88 (0.59 - 1.32) | 0.89 (0.59 - 1.34) | 0.86 (0.57 - 1.29) | 0.78 (0.50 - 1.22) |
| Only SGA | 1.35 (1.05 - 1.75) | 1.41 (1.09 - 1.83) | 1.37 (1.05 - 1.79) | 1.22 (0.93 - 1.61) | 1.14 (0.86 - 1.50) | 1.08 (0.80 - 1.46) |
| SGA and Stunted | 1.86 (1.21 - 2.84) | 2.08 (1.35 - 3.21) | 2.22 (1.41 - 3.49) | 1.95 (1.23 - 3.10) | 1.81 (1.13 - 2.88) | 2.18 (1.34 - 3.53) |
| Model 1 adjusted for sex. Model 2 = Model 1 + skin color, mother’s age at birth. Model 3 = Model 2 + maternal schooling and family income at birth. Model 4 = Model 3 + previous gestations, pregnancy risk factors, C-section, smoking in pregnancy. Model 5 = Model 4 + assets index, mother ‘nerve’ problems, father live together and history of psychiatric illness, parent’s alcoholism and breastfeeding. OR: odds ratio. SD: standard deviation. SGA: small for gestational age. NA: not applicable | | | | | | |
